# Long-term limb salvage and functional recovery in advanced Charcot neuroarthropathy complicated by a non-healing diabetic foot ulcer: a CARE-compliant case report

**DOI:** 10.3389/fendo.2026.1952152

**Published:** 2026-09-30

**Authors:** Preethi Mohan, Rajmohan Velayudhan Pillai

**Affiliations:** 1 Department of Agadatantra, Amrita Vishwa Vidyapeetham, Amrita School of Ayurveda, Kollam, India; 2 Government Ayurveda Medical College, Thiruvananthapuram, India

**Keywords:** ayurveda, case report, Charcot neuroarthropathy, diabetic foot ulcer, diabetic peripheral neuropathy, integrative medicine, limb preservation, wound healing

## Abstract

**Background:**

Advanced Charcot neuroarthropathy complicated by diabetic foot ulceration is a limb-threatening condition associated with progressive foot deformity, recurrent ulceration, infection, impaired wound healing, and a high risk of major amputation. Evidence describing long-term limb preservation following multimodal conservative management remains limited.

**Case presentation:**

A 43-year-old man with poorly controlled type 2 diabetes mellitus and diabetic peripheral neuropathy presented with advanced Charcot neuroarthropathy of the left foot, complicated by a chronic, non-healing dorsal diabetic foot ulcer (3 × 4 cm; Wagner Grade 3; University of Texas Grade 3-D) and a lateral draining sinus. During the earlier course of the disease, magnetic resonance imaging demonstrated extensive Charcot-related bony destruction with findings suggestive of secondary osteomyelitis. The diagnosis was made by the treating podiatric surgeon based on clinical and imaging findings. Microbiological and histopathological confirmation was not available. Despite prior hospitalization, incision and drainage, and approximately 15 days of intravenous antibiotic therapy, satisfactory wound healing was not achieved.

**Intervention:**

The patient underwent multimodal conservative management comprising continuation of conventional antidiabetic therapy, complete bed rest during the active wound-management period, structured local wound care using Aragvadhadi Kashayam, internal Ayurvedic medications (Pachanamritham Kashayam and Chandraprabha Vati), dietary regulation, and regular clinical follow-up. Custom-made protective footwear had previously been prescribed for the Charcot-related deformity and was used for approximately six months before discontinuation because of discomfort.

**Outcomes:**

Progressive reduction in wound exudate and slough was followed by healthy granulation tissue formation, complete epithelialization of the dorsal ulcer, and healing of the lateral draining sinus. Limb preservation was achieved without major amputation over approximately four years of follow-up. The patient subsequently regained independent ambulation, resumed routine occupational and daily activities, and maintained functional mobility. The clinical course was documented through serial clinical photographs and follow-up assessments.

**Conclusion:**

This case describes a favorable long-term outcome, including limb preservation and functional recovery, following multimodal conservative management in a patient with advanced Charcot neuroarthropathy complicated by chronic ulceration. Given the single-case design and the simultaneous use of multiple therapeutic interventions, the independent contribution of individual treatment components cannot be determined. Further prospective studies are warranted.

## 1 Introduction

Diabetic foot ulcer (DFU) is one of the most debilitating complications of diabetes mellitus and remains a leading cause of hospitalization, disability, and non-traumatic lower-extremity amputation worldwide. Despite advances in multidisciplinary care, infected and non-healing ulcers continue to impose substantial clinical, psychosocial, and economic burdens on patients and healthcare systems. Recent epidemiological evidence indicates that patients with DFUs remain at considerable risk of major amputation, particularly when ulcers are complicated by infection, neuropathy, or structural deformity (1–3).

Among the most devastating complications of diabetic peripheral neuropathy, Charcot neuroarthropathy (CN) is characterized by progressive destruction of bones and joints) represents a particularly challenging condition characterized by progressive destruction of bones and joints secondary to peripheral neuropathy. Delayed diagnosis or inadequate off-loading may result in severe deformity, recurrent ulceration, deep infection, and ultimately limb loss. The coexistence of Charcot deformity with a complicated diabetic foot ulcer substantially increases treatment complexity because successful management requires not only wound healing but also preservation of foot architecture, prevention of recurrent ulceration, and restoration of functional ambulation (4–7).

Current management of advanced Charcot neuroarthropathy emphasizes prompt diagnosis, meticulous wound care, effective infection control, strict off-loading, optimization of glycemic status, and multidisciplinary follow-up. Conservative management remains the preferred initial approach for many patients, while surgical intervention is reserved for selected cases with severe instability, deformity, or failure of conservative treatment. Nevertheless, prolonged treatment duration, recurrent ulceration, and progression to major amputation continue to be significant challenges, particularly in patients presenting with chronic non-healing wounds (8, 9).

Although numerous studies have described different approaches to diabetic foot management, well-documented reports demonstrating long-term limb preservation with sustained functional recovery in advanced Charcot neuroarthropathy remain limited, particularly following conservative management with prolonged follow-up. We therefore present a patient with advanced Charcot neuroarthropathy complicated by a chronic non-healing diabetic foot ulcer who achieved complete wound healing, long-term limb preservation, restoration of independent ambulation, and sustained functional recovery over four years following an integrative conservative management strategy. This report is presented in accordance with the CARE guidelines.

## 2 Case presentation

### 2.1 Patient information

A 43-year-old male accountant presented to our Ayurveda hospital with a chronic non-healing ulcer over the dorsal aspect of the left forefoot. His main concerns were persistent wound discharge and failure of satisfactory wound healing despite previous treatment, in the context of established Charcot neuroarthropathy. The ulcer had progressively worsened over the preceding month. He was a known case of type 2 diabetes mellitus and was receiving oral antidiabetic medication (glipizide 5 mg plus metformin 500 mg twice daily). The patient had a documented history of diabetic peripheral neuropathy and Charcot neuroarthropathy involving the left foot. Custom-made protective footwear had previously been prescribed to accommodate and protect the affected foot, with long-term use advised. The patient reported using the footwear for approximately six months but subsequently discontinued its use because of discomfort.

Before presentation to our institution, the patient had undergone previous treatment for the affected foot, including conservative management for Charcot neuroarthropathy and, during the subsequent complicated foot infection, incision and drainage of a foot abscess followed by approximately 15 days of intravenous piperacillin–tazobactam therapy. Despite these interventions, persistent wound discharge and unsatisfactory healing continued, prompting him to seek further treatment with the objective of limb preservation.

He reported persistent numbness over the affected foot, consistent with diabetic peripheral neuropathy. His body mass index was 28.4 kg/m². He had a family history of diabetes mellitus but no history of hypertension, chronic kidney disease, previous lower-limb amputation, or other major comorbidities.

### 2.2 Clinical history

The patient was diagnosed with type 2 diabetes mellitus four years before presentation but was initially non-adherent to antidiabetic medication and lifestyle modifications. In October 2020, he sustained a minor burn injury to the left foot, which subsequently progressed to infection and ulceration. Magnetic resonance imaging performed on 05 October 2020 revealed sclerosis and destruction involving the distal calcaneum, cuboid, and lateral navicular bone, accompanied by marked midfoot edema and sinus formation, with findings suggestive of Charcot neuroarthropathy complicated by secondary osteomyelitis. Computed tomography also demonstrated features consistent with Charcot-related bony destruction. The diagnosis was made by the treating podiatric surgeon based on the available clinical and imaging assessment. Microbiological confirmation through deep-tissue or bone culture and histopathological confirmation through bone biopsy were not available in the records accessible to the authors.

Following the diagnosis, conservative management comprising antimicrobial therapy, oral antidiabetic medication, and protective off-loading measures was initiated. Custom-made protective footwear was subsequently prescribed, with long-term use advised. However, the patient reported using the footwear for approximately six months before discontinuing it because of discomfort.

In early 2024, following trauma to the toenail of the affected foot, he developed progressive swelling associated with cellulitis, skin breakdown, abscess formation, and a chronic non-healing dorsal foot ulcer. He was admitted to a tertiary-care hospital, where the abscess was managed with incision and drainage, followed by approximately 15 days of intravenous piperacillin–tazobactam, a broad-spectrum antipseudomonal penicillin/β-lactamase inhibitor combination. Additional treatment included oral cefuroxime axetil, trypsin–chymotrypsin, cetirizine, multivitamins, topical wound care, and oxygen therapy.

Information regarding wound or deep-tissue bacterial culture results and the specific pathogen(s) targeted during the preceding antimicrobial therapy was not available in the clinical records accessible to the authors. Despite these interventions, wound discharge persisted, and satisfactory healing was not achieved. Given the progressive non-healing ulcer and the recommendation for major amputation, the patient sought treatment at our Ayurveda hospital with the objective of limb preservation.

### 2.3 Clinical findings

On admission, the patient was hemodynamically stable and in no acute distress. Clinical examination revealed a 3 × 4 cm ulcer over the dorsal aspect of the left forefoot, extending from the toe tips to the metatarsophalangeal joints of the second, third, and fourth toes (**Figure 1A**). The ulcer was classified as Wagner Grade 3 and University of Texas Grade 3-D.

**Figure 1:**
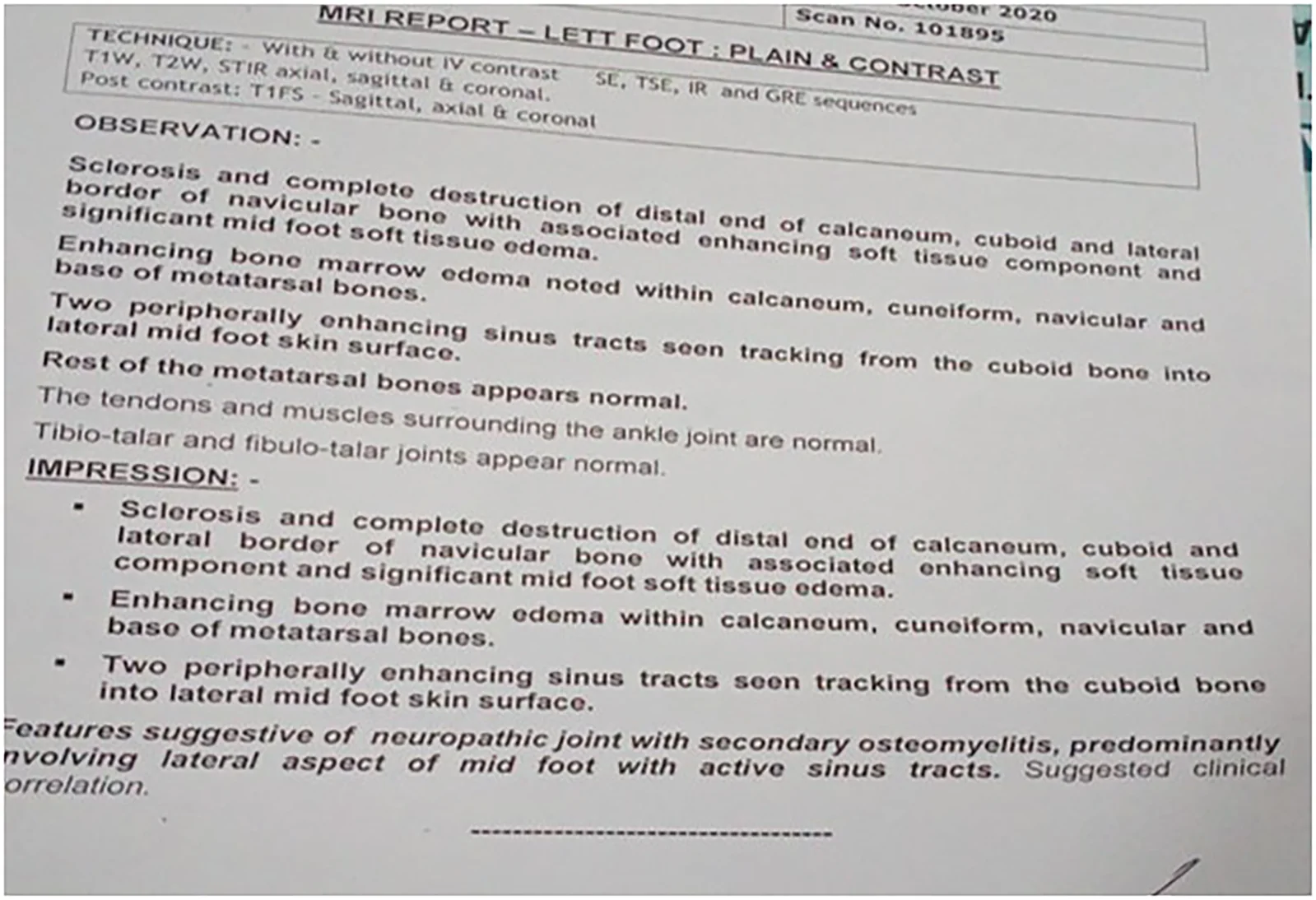
Magnetic resonance imaging (MRI) report of the left foot. MRI performed on 5 October 2020 demonstrated sclerosis and destruction of the distal calcaneum, cuboid, and lateral border of the navicular bone, with associated soft-tissue edema and bone marrow edema involving the calcaneum, cuneiform, navicular, and base of the metatarsal bones. Two sinus tracts extending from the cuboid to the lateral midfoot skin surface were identified. The radiological impression was features suggestive of a neuropathic (Charcot) joint with secondary osteomyelitis involving the lateral aspect of the midfoot and active sinus tracts.

More than 80% of the wound bed was covered with thick yellowish-white slough and necrotic tissue, with profuse purulent exudate associated with a foul odor. The wound margins were macerated, and the surrounding skin was erythematous and edematous, consistent with surrounding cellulitis. The dorsum of the foot exhibited xerosis with fissuring, consistent with diabetic autonomic neuropathy. A localized violaceous discoloration of the surrounding skin was also observed, raising concern regarding compromised local tissue perfusion. Diffuse swelling of the affected foot and deformity consistent with established Charcot neuroarthropathy were present.

Peripheral arterial pulsations over the dorsalis pedis and posterior tibial arteries were palpable. Overall, the patient presented with a severe infected diabetic foot ulcer characterized by extensive slough and necrotic tissue, profuse foul-smelling purulent discharge, surrounding cellulitis, and underlying Charcot-related foot deformity.

### 2.4 Diagnostic assessment

The diagnostic assessment included detailed clinical evaluation, laboratory investigations, radiological imaging, and Ayurvedic clinical assessment to evaluate the severity of the condition, identify associated complications, and guide subsequent management.

The diagnosis of advanced Charcot neuroarthropathy was supported by the patient’s history of diabetic peripheral neuropathy, established foot deformity, and radiological evidence of extensive bony destruction and joint disorganization. The presence of a deep diabetic foot ulcer with purulent discharge, surrounding cellulitis, and a lateral draining sinus indicated a severe complicated diabetic foot infection.

A diagnostic challenge was the interpretation of possible superimposed osteomyelitis in the presence of extensive Charcot-related osseous destruction and bone marrow abnormalities. The MRI performed in 2020 demonstrated extensive Charcot-related bony destruction, marrow oedema, sinus formation, and findings suggestive of secondary osteomyelitis. However, bone biopsy, histopathological examination, and deep-tissue or bone culture results were not available. Therefore, secondary osteomyelitis was considered a suspected complication based on the available clinical and radiological findings and was not microbiologically or histopathologically confirmed.

#### 2.4.1 Hematological investigations

Baseline laboratory investigations showed a hemoglobin level of 12.0 g/dL and a total leukocyte count of 7,600/mm³, comprising 51% neutrophils, 45% lymphocytes, 3% eosinophils, 1% monocytes, and no basophils. Inflammatory markers were elevated, with an erythrocyte sedimentation rate (ESR) of 57 mm/hour and a C-reactive protein (CRP) level of 12.98 mg/L, indicating an ongoing inflammatory process. The complete laboratory profile, including hematological, glycemic, renal, and biochemical parameters, is presented in **Table 1**.

**Table 1:** Baseline laboratory investigations.

Parameter	Patient value	Reference range
Hematology		
Hemoglobin	12.0 g/dL	13.0–18.0 g/dL (Male)
Total leukocyte count	7,600/mm³	4,000–11,000/mm³
Neutrophils	51%	40–75%
Lymphocytes	45%	20–45%
Eosinophils	3%	0–6%
Monocytes	1%	1–7%
Basophils	0%	0–1%
ESR	57 mm/h	0–15 mm/h
CRP	12.98 mg/L	<5.0 mg/L
Glycemic profile		
HbA1c	9.0%	4.2–6.0%
Renal profile		
Blood urea	24 mg/dL	10–40 mg/dL
Serum creatinine	0.8 mg/dL	0.5–1.3 mg/dL
Uric acid	6.5 mg/dL	3.4–7.0 mg/dL (Male)
Sodium	130 mmol/L	135–145 mmol/L
Potassium	4.47 mmol/L	3.5–5.1 mmol/L
Chloride	98.2 mmol/L	98–107 mmol/L
Liver function tests		
Total bilirubin	0.4 mg/dL	≤1.2 mg/dL
Direct bilirubin	0.2 mg/dL	<0.3 mg/dL
Indirect bilirubin	0.2 mg/dL	0.2–0.9 mg/dL
AST	11 IU/L	0–40 IU/L
ALT	12 IU/L	0–41 IU/L
Alkaline phosphatase	91 IU/L	35–129 IU/L
GGT	31 U/L	6–71 U/L
Total protein	7.2 g/dL	6.6–8.7 g/dL
Albumin	4.0 g/dL	3.5–5.2 g/dL
Globulin	3.1 g/dL	2.3–3.5 g/dL
Albumin/Globulin ratio	1.3	1.0–2.2

#### 2.4.2 Renal function

Baseline renal function was preserved, with blood urea and serum creatinine levels within the normal reference range. Mild hyponatremia (serum sodium, 130 mmol/L) was noted at presentation. Relevant renal and biochemical parameters are summarized in **Table 1**.

#### 2.4.3 Liver function

Baseline liver function tests, including serum bilirubin, transaminases, alkaline phosphatase, gamma-glutamyl transferase, serum proteins, and the albumin–globulin ratio, were within the normal reference ranges (**Table 1**).

#### 2.4.4 Urine examination

Routine urine examination was unremarkable, with no evidence of glucosuria, proteinuria, red blood cells, pus cells, or epithelial cells.

#### 2.4.5 Wound assessment

Detailed wound assessment revealed a 3 × 4 cm ulcer over the dorsal aspect of the left forefoot, extending from the tips of the toes to the metatarsophalangeal joints of the second, third, and fourth toes. The ulcer was classified as Wagner Grade 3 and University of Texas Grade 3-D.

More than 80% of the wound bed was covered by thick yellowish-white slough and necrotic tissue, with less than 20% of the wound bed covered by healthy granulation tissue at baseline (**Figure 2**). Profuse purulent exudate with a foul odor was present, and the wound margins were macerated. The surrounding skin was erythematous and edematous, consistent with cellulitis (**Figure 1**).

**Figure 2:**
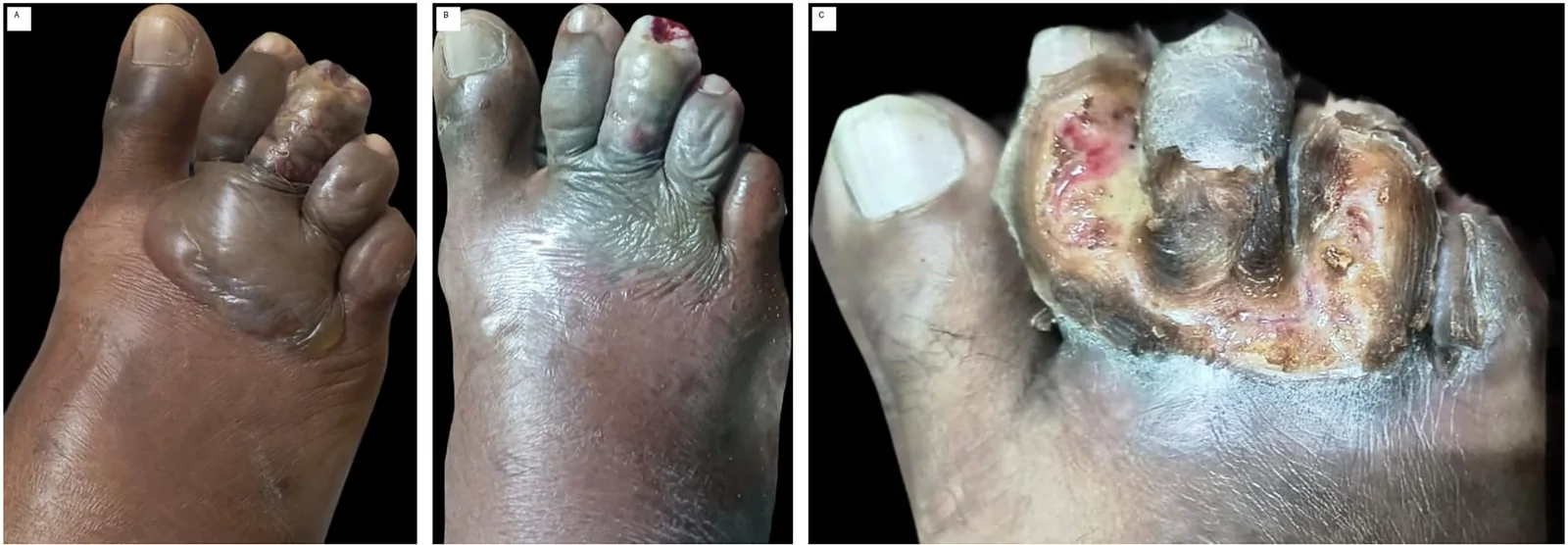
Clinical progression of the affected foot prior to treatment. **(A)** Early stage showing diffuse swelling of the left forefoot with a tense hemorrhagic blister over the dorsum, consistent with acute soft-tissue involvement associated with Charcot neuroarthropathy. **(B)** Progression of the lesion with rupture of the blister, increasing edema, skin discoloration, and early soft-tissue breakdown. **(C)** Established dorsal diabetic foot ulcer with extensive tissue loss, slough, necrotic tissue, and exposure of deep tissues before initiation of treatment.

A draining sinus was present over the lateral aspect of the foot. The sinus was located in the region corresponding to the sinus tract described on the previous MRI examination. A plantar callosity was also noted at baseline. The affected foot demonstrated diffuse swelling and deformity consistent with chronic Charcot neuroarthropathy. Baseline wound characteristics are summarized in **Table 2**.

**Table 2:** Baseline wound assessment.

Parameter	Finding
Ulcer location	Dorsal aspect of the left forefoot
Ulcer size	3 × 4 cm
Wagner classification	Grade 3
University of Texas classification	Grade 3-D
Wound bed	>80% slough and necrotic tissue
Healthy granulation tissue	<20%
Wound margins	Macerated
Exudate	Profuse purulent
Odor	Foul odor present
Periwound cellulitis	Present
Lateral draining sinus	Present
Plantar callosity	Present
Foot swelling	Diffuse swelling present
Charcot deformity	Present

#### 2.4.6 Neurological assessment

Neurological examination was consistent with diabetic peripheral neuropathy. Assessment of the affected (left) foot using the 10-g Semmes–Weinstein monofilament was not performed because of the extensive dorsal ulcer, active infection, and plantar callosities, which could have interfered with accurate sensory assessment. As monofilament testing could not be reliably performed on the ulcerated foot, sensory assessment was performed on the contralateral foot, together with vibration perception, pin-prick sensation, deep tendon reflexes, and the Neuropathy Disability Score (NDS), to characterize the patient’s underlying diabetic peripheral neuropathy.

The contralateral (right) foot was evaluated at four standard plantar sites using the 10-g Semmes–Weinstein monofilament. Protective sensation was absent at the distal hallux and diminished over the first, third, and fifth metatarsal heads, indicating loss of protective sensation (LOPS). Vibration perception and pin-prick sensation were impaired. Deep tendon reflex examination demonstrated diminished ankle jerks with preserved knee jerks. The Neuropathy Disability Score (NDS) was 7.5, consistent with moderate diabetic peripheral neuropathy.

#### 2.4.7 Radiological assessment

Plain radiographs of the affected (left) foot demonstrated features consistent with chronic Charcot neuroarthropathy, including bony destruction, joint disorganization, and midfoot deformity.

Magnetic resonance imaging performed on 05 October 2020, during the earlier course of the patient’s disease, demonstrated extensive destruction involving the distal calcaneum, cuboid, and lateral navicular bone, associated with diffuse bone marrow edema. The MRI also demonstrated sinus tract formation from the cuboid region with surrounding soft-tissue edema and findings suggestive of secondary osteomyelitis (**Figure 1**).

These imaging findings formed part of the clinical and radiological assessment on which the treating podiatric surgeon based the diagnosis during the earlier disease course. Microbiological confirmation through deep-tissue or bone culture and histopathological confirmation through bone biopsy were not available in the clinical records accessible to the authors.

The MRI findings are therefore presented as evidence suggestive of secondary osteomyelitis during the earlier disease course and should not be interpreted as microbiological or histopathological confirmation of osteomyelitis during the 2024 ulcer episode.

#### 2.4.8 Final diagnosis

The final diagnosis at presentation to our center was advanced Charcot neuroarthropathy of the left foot complicated by a severe non-healing diabetic foot ulcer (Wagner Grade 3; University of Texas Grade 3-D) in a patient with poorly controlled type 2 diabetes mellitus and diabetic peripheral neuropathy.

The patient had a previous clinical and radiological diagnosis of suspected secondary osteomyelitis during the earlier course of Charcot neuroarthropathy. MRI performed in October 2020 demonstrated extensive Charcot-related bony destruction, bone marrow edema, sinus tract formation, and findings suggestive of secondary osteomyelitis. Microbiological and histopathological confirmation of osteomyelitis was not available.

### 2.5 Therapeutic intervention

#### 2.5.1 Previous conventional management

Before presentation to our institution, the patient had been managed at a tertiary-care hospital for a complicated diabetic foot infection. A localized abscess involving the affected foot was treated with incision and drainage, followed by approximately 15 days of intravenous piperacillin–tazobactam, a broad-spectrum antipseudomonal penicillin/β-lactamase inhibitor combination. Additional treatment included oral cefuroxime axetil, trypsin–chymotrypsin, cetirizine, multivitamins, topical wound care, and oxygen therapy.

Despite these interventions, the wound continued to exhibit persistent purulent discharge with unsatisfactory healing. Information regarding wound or deep-tissue bacterial culture results and the specific pathogen(s) targeted during the preceding antimicrobial therapy was not available in the clinical records accessible to the authors. Owing to the extensive tissue involvement and poor clinical response, major amputation was advised. Seeking an alternative approach for limb preservation, the patient subsequently presented to our Ayurveda hospital.

The patient continued his previously prescribed oral antidiabetic medications, comprising glipizide and metformin, throughout the treatment period without modification.

Before presentation to our institution, custom protective footwear had been prescribed for the Charcot-related foot deformity, with long-term use advised. The patient reported using the footwear for approximately six months before discontinuing it because of discomfort. During the active treatment period at our institution, the patient was maintained on complete bed rest as part of the management strategy for the active wound.

#### 2.5.2 Ayurvedic clinical assessment

From an Ayurvedic perspective, the clinical presentation was diagnosed as *Madhumehajanya Duṣṭa Vraṇa*, a chronic non-healing ulcer associated with *Madhumeha* (diabetes mellitus). According to Ayurvedic principles, the condition is understood to involve impaired tissue healing associated with pathological interactions between *doṣas* and *duṣyas*, contributing to delayed wound repair and chronic ulceration.

According to the principles described in the *Suśruta Saṃhitā*, the management of *Duṣṭa Vraṇa* follows a sequential therapeutic approach consisting of *Śodhana* (wound cleansing and preparation of the wound bed) followed by *Ropaṇa* (promotion of wound healing and tissue repair). These principles formed the Ayurvedic therapeutic framework for the management strategy adopted in the present case.

#### 2.5.3 Local wound management

Local wound management consisted of twice-daily cleansing, irrigation, and soaking with *Aragvadhādi Kaṣāya* throughout the active wound-healing period. This regimen was continued until complete epithelialization of the ulcer.

Within the Ayurvedic therapeutic framework, the primary objective of *Śodhana* was wound cleansing and preparation of the wound bed. The local regimen was intended to facilitate the removal of slough and devitalized tissue, reduce excessive wound exudate, maintain wound hygiene, and prepare the wound bed for subsequent healing.

Following complete epithelialization, *Murivenna*, a classical Ayurvedic medicated oil traditionally used in wound care, was applied externally to the healed area to support residual skin recovery and scar maturation.

#### 2.5.4 Internal Ayurvedic management

Internal Ayurvedic treatment consisted of *Pachanamritham Kaṣāyam* and *Chandraprabha Vaṭi*, administered as part of the treatment regimen. These formulations were selected according to the Ayurvedic diagnosis of *Madhumehajanya Duṣṭa Vraṇa*.

Within the Ayurvedic therapeutic framework, the treatment objectives included addressing the underlying disease process and supporting wound healing and tissue repair. The patient continued his prescribed conventional oral antidiabetic medications without modification throughout the course of Ayurvedic treatment.

The therapeutic approach was modified according to the stage of wound healing. During the active wound phase, local cleansing, irrigation, and soaking with Aragvadhādi Kaṣāya were continued together with internal Ayurvedic medications and complete bed rest. Following complete epithelialization of the dorsal ulcer and lateral draining sinus, active wound-cleansing procedures were discontinued, and Murivenna was subsequently introduced for external application to support local skin care and scar maturation.

Details regarding the individual Ayurvedic formulations, including the dose, route, frequency, duration, composition, and source of the preparations, are provided in **Table 3**. All Ayurvedic medicines used in the present case were obtained from a GMP-certified pharmacy.

**Table 3:** Therapeutic intervention.

Intervention	Dose/Frequency	Duration	Purpose
Aragvadhādi Kaṣāya (local cleansing, irrigation, and soaking)	Twice daily	Until complete epithelialization	*Śodhana* (local wound cleansing and preparation of the wound bed)
Pachanamritham Kaṣāyam	45 mL twice daily	Until complete epithelialization	Internal Ayurvedic management
Chandraprabha Vaṭ?i	2 tablets twice daily (2-0-2)	Until complete epithelialization	Internal Ayurvedic management
Murivenna (external application)	Applied externally as required	After complete epithelialization	Scar maturation and improvement of residual discoloration
Conventional antidiabetic medications	Continued unchanged	Throughout treatment	Glycemic management
Complete bed rest	Continuous during the active wound-management period	During the active treatment period, until clinically appropriate mobilization	Avoidance of mechanical loading of the affected foot during active wound management

Conventional oral antidiabetic therapy was continued throughout the treatment period without modification. Murivenna was introduced only after the wounds had completely epithelialized.

### 2.6 Clinical course

Following initiation of the multimodal conservative management strategy, the patient demonstrated progressive clinical improvement (**Table 4**; **Figures 3**, **4**). During the first week, purulent discharge, foul odor, surrounding oedema, and local inflammatory changes decreased substantially, resulting in a cleaner wound bed. By the second week, the amount of slough had reduced considerably, with the development of healthy granulation tissue over both the dorsal ulcer and the lateral draining sinus.

**Table 4:** Timeline of clinical outcomes.

Time point	Clinical findings/Management/Outcome
October 2020	The patient sustained a minor burn injury to the left foot, followed by infection and ulceration.
05 October 2020	MRI demonstrated extensive Charcot-related bony destruction involving the distal calcaneum, cuboid, and lateral navicular bone, with marked midfoot oedema and sinus formation. The imaging findings were suggestive of secondary osteomyelitis.
2020–2024	Conservative management for Charcot neuroarthropathy was continued. Protective/off-loading footwear was advised; however, the patient reportedly used it for approximately six months and subsequently discontinued it because of discomfort.
Early 2024	Following trauma to the toenail of the affected foot, the patient developed progressive swelling, cellulitis, skin breakdown, abscess formation, and a chronic non-healing dorsal diabetic foot ulcer.
Before presentation to our institution (2024)	The foot abscess was managed at a tertiary-care hospital with incision and drainage followed by approximately 15 days of intravenous piperacillin–tazobactam therapy. Despite treatment, persistent wound discharge and unsatisfactory healing continued, and major amputation was advised.
Baseline (presentation to our institution)	A 3 × 4 cm dorsal diabetic foot ulcer (Wagner Grade 3; University of Texas Grade 3-D) with extensive slough and necrotic tissue, profuse purulent discharge, foul odor, surrounding cellulitis, an active lateral draining sinus, and established Charcot deformity was observed.
Week 1	Reduction in purulent wound discharge, foul odor, surrounding oedema, and local inflammation, with progressive cleaning of the wound bed.
Week 2	Further reduction in slough with development of healthy granulation tissue over both the dorsal ulcer and lateral draining sinus.
Week 3	Progressive wound contraction and advancing epithelialization of both the dorsal ulcer and lateral draining sinus.
Week 4	Complete epithelialization of the dorsal ulcer and lateral draining sinus, with no further discharge.
Post-healing phase	Active wound-cleansing procedures were discontinued. *Murivenna* was introduced as an external application to support local skin care, scar maturation, and improvement of residual discoloration. The patient gradually resumed ambulation following wound healing and clinical stabilization.
Approximately four-year follow-up	Sustained limb preservation with no recurrence of ulceration or sinus formation and no requirement for major amputation. Independent ambulation and routine occupational and daily activities were maintained despite the persistent Charcot deformity.

**Figure 3:**
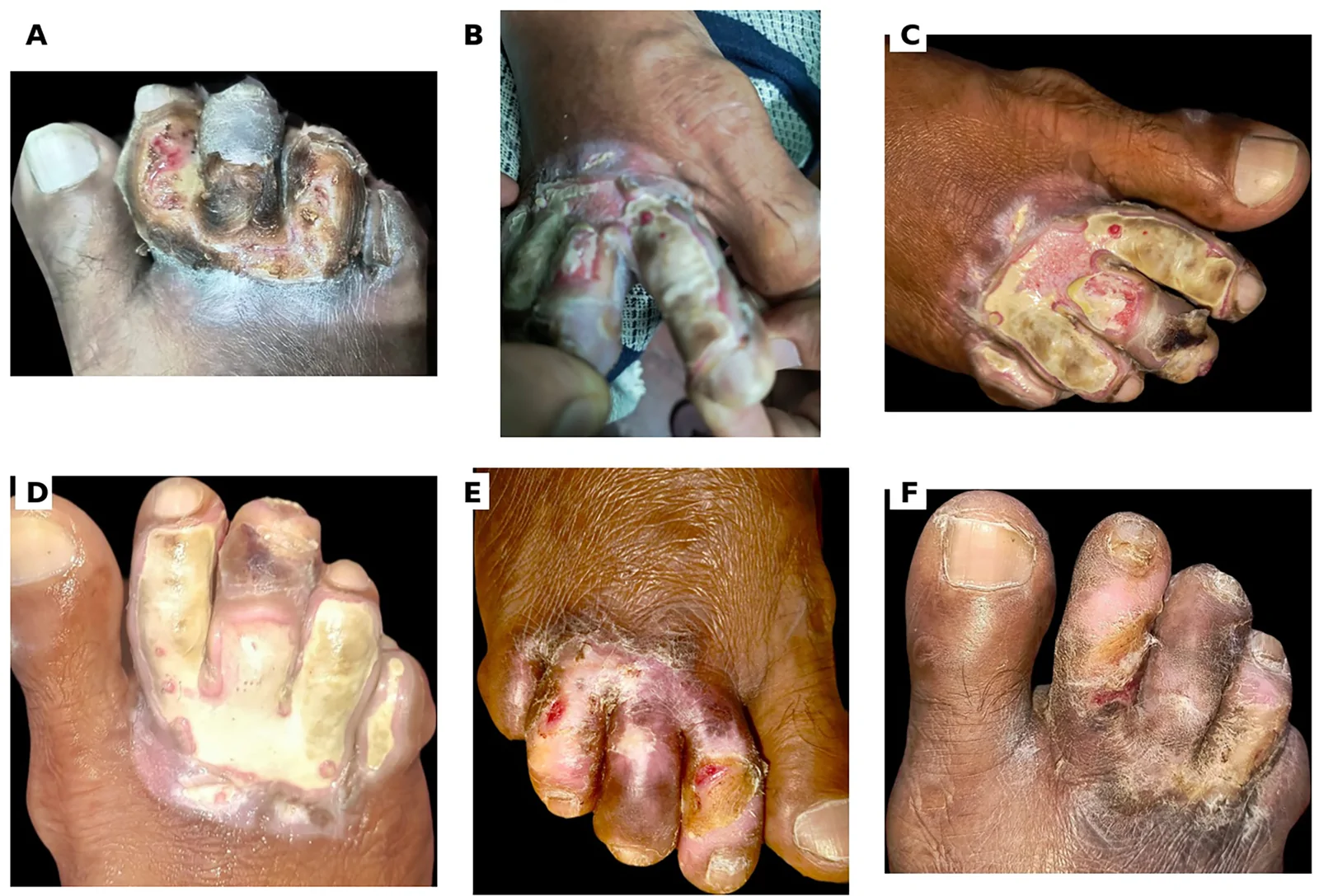
Sequential healing of the dorsal diabetic foot ulcer following integrative management. **(A)** Baseline ulcer before treatment demonstrating extensive slough, necrotic tissue, surrounding edema, and active infection. **(B)** Early reduction in slough and exudate with the appearance of healthy granulation tissue. **(C)** Progressive wound contraction with continued granulation tissue formation. **(D)** Advanced epithelialization with marked reduction in ulcer size. **(E)** Near-complete epithelialization with restoration of skin continuity. **(F)** Complete epithelialization of the dorsal ulcer without clinical evidence of active infection.

**Figure 4:**
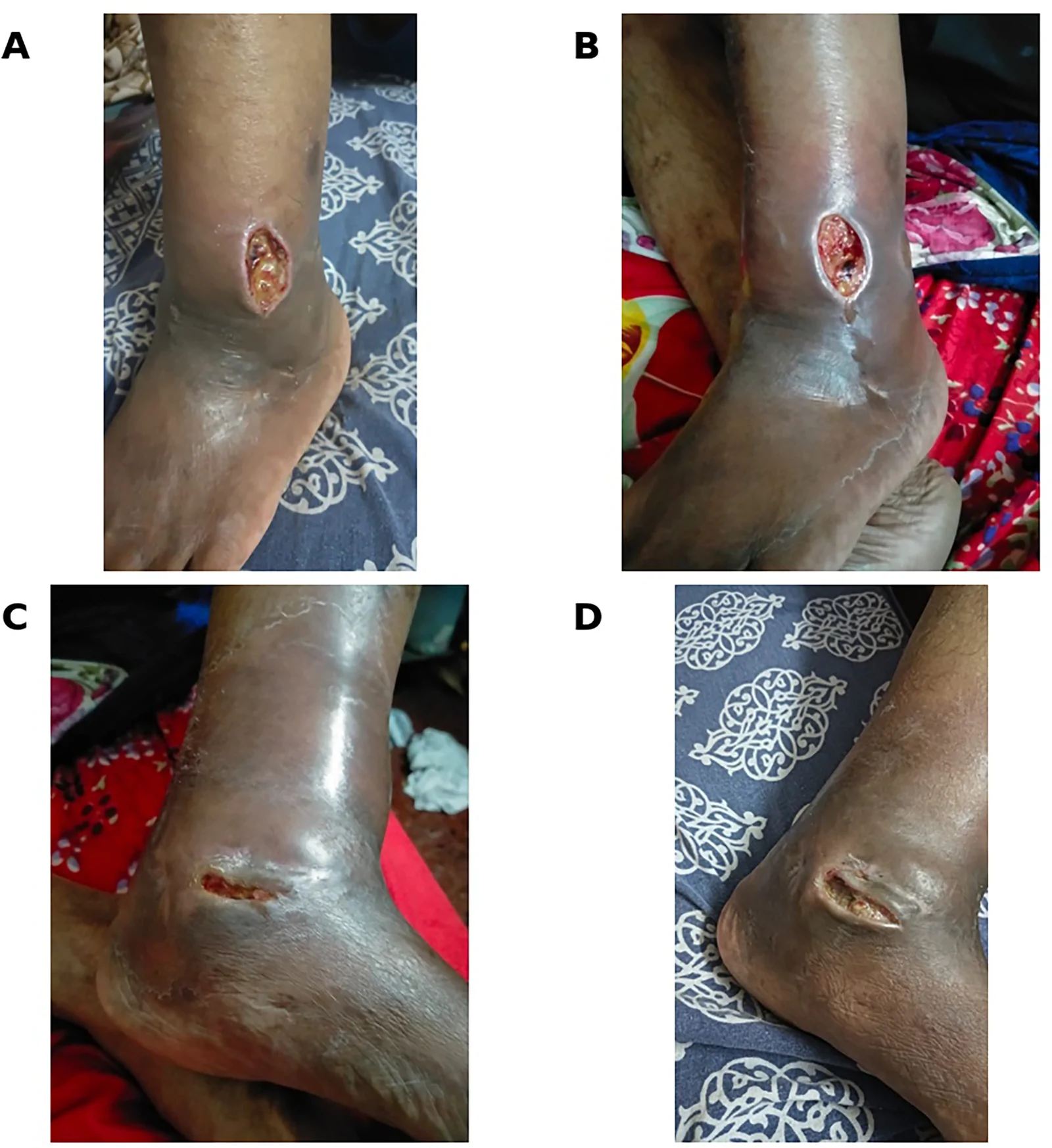
Sequential healing of the lateral draining sinus following integrative management. **(A)** Baseline lateral draining sinus before treatment demonstrating an active sinus opening with slough. **(B)** Early healing with reduction in slough and development of healthy granulation tissue. **(C)** Progressive wound contraction with advancing epithelialization. **(D)** Complete epithelialization of the lateral sinus with restoration of skin integrity and no clinical evidence of active drainage.

Over the subsequent weeks, complete epithelialization of both the dorsal ulcer and the lateral draining sinus was achieved without further discharge (**Figures 3**, **4**). Following wound closure, *Murivenna* was applied externally to support local skin care and scar maturation. With continued treatment and follow-up, progressive wound contraction, reduction in exudate, and advancing epithelialization were observed.

During the active wound-management period, the patient remained on complete bed rest to avoid mechanical loading of the affected foot. Following wound healing and clinical stabilization, gradual mobilization was undertaken as clinically appropriate.

At long-term follow-up, approximately four years after treatment, the patient remained free of ulcer recurrence or sinus formation, had preserved the affected limb, regained independent ambulation, and resumed routine daily activities.

### 2.7 Follow-up and outcomes

The patient was followed up regularly after completion of treatment. Complete epithelialization of both the dorsal diabetic foot ulcer and the lateral draining sinus was achieved, with no recurrence of ulceration, sinus formation, or clinically documented local infection during approximately four years of follow-up. During the active wound-management period, the patient maintained complete bed rest. At long-term follow-up, he had regained independent ambulation, with substantial improvement in functional mobility, enabling him to resume his routine occupational and daily activities. No additional surgical intervention or major amputation was required during follow-up. At the most recent follow-up, the patient completed the Sabarimala pilgrimage, demonstrating sustained ambulatory function and long-term limb preservation despite advanced Charcot neuroarthropathy.

Follow-up assessments included serial clinical evaluation of wound closure, wound discharge, sinus formation, local signs of infection, limb preservation, and ambulatory function. Clinician-assessed outcomes demonstrated complete epithelialization of both the dorsal ulcer and lateral draining sinus, with no recurrence of ulceration, sinus formation, or clinically evident local infection during approximately four years of follow-up. Patient-assessed functional recovery was reflected by the return to independent ambulation and routine occupational and daily activities. At the most recent follow-up, the patient reported sustained ambulatory function and successful completion of the Sabarimala pilgrimage.

The patient reported adherence to the prescribed treatment regimen during the active treatment period. No treatment-related adverse or unanticipated events were reported during the documented course of treatment and follow-up.

## 3 Discussion

### 3.1 Clinical significance of Charcot neuroarthropathy

Charcot neuroarthropathy (CN) is one of the most severe complications associated with diabetic peripheral neuropathy and is an important cause of lower-extremity morbidity and limb loss among individuals with diabetes. The coexistence of advanced Charcot deformity and chronic diabetic foot ulceration presents a substantial therapeutic challenge, often requiring prolonged multidisciplinary care and, in severe cases, major amputation. Consequently, successful long-term limb preservation in such patients is of considerable clinical interest (10–12).

CN is characterized by progressive destruction of bones, joints, and surrounding soft tissues resulting from repetitive mechanical trauma to an insensate foot. Delayed diagnosis and continued mechanical stress during the active phase may contribute to progressive deformity, recurrent ulceration, deep infection, and an increased risk of major amputation. Accordingly, conservative management requires attention to mechanical protection of the affected foot, meticulous wound care, appropriate infection management, optimization of glycaemic status, and long-term surveillance to preserve limb function and prevent further complications (13–20).

The present case involved advanced Charcot neuroarthropathy complicated by a chronic non-healing diabetic foot ulcer and persistent wound discharge. The patient also had previous MRI findings from 2020 that were suggestive of secondary osteomyelitis. Before presentation to our institution in 2024, he had undergone hospitalization, incision and drainage, and approximately 15 days of intravenous piperacillin–tazobactam therapy without satisfactory wound healing, and major amputation had been advised.

In contrast to reports limited to short-term wound outcomes, this case documents approximately four years of follow-up demonstrating complete healing of both the dorsal ulcer and the lateral draining sinus, restoration of independent ambulation, sustained functional recovery, and long-term limb preservation without recurrence. These outcomes are clinically meaningful because preservation of mobility, maintenance of functional independence, and avoidance of major amputation are important therapeutic goals in patients with advanced Charcot neuroarthropathy. Although causal inferences cannot be drawn from a single case, this report demonstrates that successful long-term limb preservation and functional recovery may be achievable in selected patients receiving comprehensive conservative management.

### 3.2 Challenges in conservative limb salvage

Limb salvage in advanced Charcot neuroarthropathy extends beyond wound closure and requires simultaneous attention to infection, mechanical protection of the affected foot, prevention of recurrent ulceration, optimization of metabolic status, and restoration of functional mobility. Chronic hyperglycaemia, peripheral neuropathy, repetitive mechanical stress, impaired wound healing, and progressive foot deformity interact to create a complex clinical environment associated with an increased risk of recurrent ulceration, infection, and major amputation. Consequently, successful conservative management requires coordinated multidisciplinary care and long-term surveillance (21–25).

This case illustrates the complexity of conservative limb salvage in advanced Charcot neuroarthropathy. The patient presented with several adverse clinical features, including advanced deformity, a chronic ulcer, a draining sinus, and a previous MRI examination with findings suggestive of secondary osteomyelitis. However, the available clinical records did not include deep-tissue or bone culture results, and bone biopsy or histopathological confirmation was not available. Therefore, the presence of osteomyelitis should be interpreted as clinically and radiologically suspected rather than definitively confirmed.

Despite these challenges, complete wound healing was achieved without major surgical intervention. The favorable long-term outcome was accompanied by restoration of independent ambulation and absence of ulcer recurrence during approximately four years of follow-up.

Although causal inferences cannot be drawn from a single case, the sustained outcome highlights the importance of comprehensive conservative management, including meticulous wound care, mechanical protection during the active wound-management period, metabolic management, infection management, and regular long-term follow-up in carefully selected patients.

### 3.3 Clinical rationale for the management approach

Management of advanced Charcot neuroarthropathy complicated by a severe diabetic foot ulcer requires simultaneous attention to local wound conditions, mechanical protection of the affected foot, metabolic control, and prevention of further tissue injury. In the present case, conventional antidiabetic therapy was continued without modification, while complete bed rest was maintained during the active wound-management period to avoid mechanical loading of the affected foot. Local and systemic Ayurvedic interventions were incorporated as components of an integrative conservative management strategy.

From an Ayurvedic perspective, the clinical presentation was interpreted as *Madhumehajanya Duṣṭa Vraṇa*, a chronic non-healing wound associated with *Madhumeha* (diabetes mellitus). Based on the principles described in the *Suśruta Saṃhitā*, treatment was planned sequentially, beginning with *Śodhana* (wound cleansing and preparation of the wound bed), followed by *Ropaṇa* (promotion of tissue healing and regeneration).

Within this therapeutic framework, local wound care with *Aragvadhādi Kaṣāya* was intended to facilitate wound cleansing and preparation of the wound bed. The internal formulations *Pachanamritham Kaṣāyam* and *Chandraprabha Vaṭi* were administered as part of the overall Ayurvedic management strategy. These interventions were provided alongside, rather than in place of, conventional antidiabetic therapy and other supportive measures.

Because multiple interventions were implemented concurrently, the contribution of any individual component to the observed outcome cannot be determined.

### 3.4 Interpretation of the observed outcomes

The clinical course observed in this case provides several clinically relevant observations; however, causal relationships cannot be established from a single case report.

First, wound healing occurred despite suboptimal glycaemic control during the treatment period. Optimization of glycaemic status remains an important component of diabetic foot management. Nevertheless, this observation illustrates the multifactorial nature of wound healing and the potential importance of coordinated management of local wound conditions, mechanical protection, infection, and metabolic factors. The present case should not be interpreted as suggesting that adequate glycaemic control is unnecessary for successful wound healing.

Second, the durability of the outcome is noteworthy. In advanced Charcot neuroarthropathy, recurrent ulceration, progressive deformity, infection, and limb loss remain important long-term concerns. The absence of ulcer recurrence, sinus formation, or clinically documented local infection and the avoidance of major amputation during approximately four years of follow-up suggest sustained clinical stability rather than short-term wound closure alone.

Accordingly, the favorable clinical outcome should be interpreted as an observation following a multimodal integrative conservative management strategy rather than evidence of efficacy of any individual therapeutic component.

Finally, this case emphasizes the importance of evaluating functional recovery in addition to anatomical wound healing. Restoration of independent ambulation, return to routine occupational and daily activities, and successful completion of the Sabarimala pilgrimage indicate meaningful long-term functional recovery from the patient’s perspective. These observations reinforce the value of incorporating functional outcomes alongside wound closure when assessing conservative limb-salvage strategies in advanced Charcot neuroarthropathy.

### 3.5 Role of dietary and lifestyle measures within the integrative approach

The integrative management strategy also incorporated Ayurvedic dietary and lifestyle recommendations alongside local wound care, internal Ayurvedic formulations, complete bed rest during the active wound-management period, and continuation of conventional diabetes therapy. The independent contribution of these components cannot be determined.

From an Ayurvedic perspective, *Pathya Āhāra* (wholesome dietary regulation) forms an integral component of the management of both *Madhumeha* and *Duṣṭa Vraṇa*. The prescribed dietary regimen emphasized foods with a lower glycaemic load while limiting highly processed foods and excessive intake of spicy, salty, and sour foods. These measures were incorporated as supportive components of the overall management strategy.

Adequate nocturnal sleep and lifestyle regulation were also advised as supportive measures according to Ayurvedic principles. The specific contribution of these lifestyle interventions to the observed clinical outcome cannot be established in the present case.

Because the patient received multiple concurrent interventions, including conventional diabetes therapy, previous antimicrobial and surgical management, bed rest, local wound care, internal Ayurvedic formulations, dietary regulation, and lifestyle measures, the observed outcome cannot be attributed to any individual intervention. The findings should therefore be interpreted as describing the clinical course following a multimodal integrative conservative management strategy rather than demonstrating the efficacy of a specific treatment component (26–28).

### 3.6 Strengths of this case

The present case has several strengths. First, advanced Charcot neuroarthropathy and wound severity were characterized using clinical assessment, radiological findings, and internationally recognized wound classification systems, including the Wagner and University of Texas classifications.

Second, serial clinical photographs documented the progression from active ulceration to complete wound healing, providing visual documentation of the clinical course.

Third, the approximately four-year follow-up demonstrated sustained limb preservation, independent ambulation, and absence of ulcer recurrence or major amputation, extending beyond short-term wound-healing outcomes commonly reported in individual cases.

Finally, the management strategy and therapeutic interventions have been described in detail, including the concurrent use of conventional antidiabetic therapy and Ayurvedic interventions, thereby improving transparency regarding the multimodal nature of the management approach (29).

### 3.7 Limitations

This report has several limitations. First, as a single-case report, the findings cannot establish treatment efficacy, causality, or generalizability.

Second, although the MRI performed in 2020 demonstrated findings suggestive of secondary osteomyelitis, deep-tissue or bone microbiological culture and histopathological confirmation were unavailable. Therefore, osteomyelitis could not be definitively confirmed, and its subsequent resolution could not be objectively established.

Third, the previous antimicrobial treatment was documented as approximately 15 days of intravenous piperacillin–tazobactam, but information regarding wound culture results and the specific pathogen(s) targeted was not available in the clinical records accessible to the authors.

Fourth, functional recovery was assessed clinically rather than using standardized patient-reported or validated functional outcome measures.

Fifth, follow-up MRI was not performed; therefore, radiological changes in Charcot neuroarthropathy and the status of the previously suspected osteomyelitis could not be evaluated.

Finally, because multiple interventions were implemented concurrently within an integrative conservative management strategy, including conventional diabetes treatment, bed rest, local wound care, internal Ayurvedic formulations, dietary measures, and lifestyle advice, the independent contribution of individual treatment components could not be determined.

Further prospective studies involving larger patient cohorts, standardized functional outcome measures, systematic microbiological assessment when clinically indicated, and serial radiological evaluation are required to evaluate the safety, reproducibility, and clinical outcomes associated with this multimodal management approach.

### 3.8 Conclusion

This case documents successful long-term limb preservation and functional recovery in a patient with advanced Charcot neuroarthropathy complicated by a severe non-healing diabetic foot ulcer. Complete wound healing, restoration of independent ambulation, and sustained functional recovery without ulcer recurrence or major amputation during approximately four years of follow-up underscore the clinical significance of the observed outcome.

The favorable clinical course occurred in the context of a multimodal conservative management strategy incorporating conventional diabetes therapy, mechanical protection through complete bed rest during the active wound-management period, local wound care, and Ayurvedic interventions. Because this report describes a single patient receiving multiple concurrent interventions, no conclusions regarding the efficacy or independent contribution of any individual treatment component can be drawn.

Further prospective studies are warranted to evaluate the safety, reproducibility, and clinical effectiveness of comprehensive conservative management strategies for limb preservation in advanced Charcot neuroarthropathy.

## 4 Patient perspective

The patient expressed satisfaction with the treatment outcome, particularly the complete healing of the ulcer, preservation of the affected limb, and restoration of independent mobility. He reported that he was able to resume his occupational and daily activities and complete the Sabarimala pilgrimage, which he regarded as a major milestone in his recovery.

## Data Availability

The original contributions presented in the study are included in the article/supplementary material. Further inquiries can be directed to the corresponding author.
